# 30 Years Old: *O*-GlcNAc Reaches the Age of Reason – Regulation of Cell Signaling and Metabolism by *O*-GlcNAcylation

**DOI:** 10.3389/fendo.2015.00017

**Published:** 2015-02-09

**Authors:** Tony Lefebvre, Tarik Issad

**Affiliations:** ^1^Structural and Functional Glycobiology Unit, CNRS-UMR 8576, Lille 1 University, Villeneuve d’Ascq, France; ^2^CNRS-UMR 8104, Institut Cochin, Université Paris Descartes, Paris, France; ^3^U1016, INSERM, Paris, France

**Keywords:** *O*-GlcNAc, cell signaling, *O*-GlcNAcylation, editorial, proteins

Hundreds of post-translational modifications (PTM) have been characterized on proteins, including a large variety of glycosylations, among which figures *O*-GlcNAcylation. Since its discovery, *O*-GlcNAcylation has emerged as a major PTM that is widespread, being found in viruses, bacteria, and protists through plant and animal cells. In contrast to *N*- and *O*-glycosylations, *O*-GlcNAcylation involves only the transfer of a single *N*-acetylglucosamine moiety through a beta-linkage onto serine and threonine residues of proteins that are localized to the cytosol, nucleus, and mitochondria. The *O*-GlcNAc group is provided by UDP–GlcNAc, the end-product of the hexosamine biosynthetic pathway (HBP), which integrates several metabolic pathways. *O*-GlcNAcylation levels therefore tightly depend on the nutritional status; regulation of functions by this PTM is thus intimately linked to lifestyle and environment (1, 2). As with phosphorylation, with which it can compete, *O*-GlcNAcylation is reversible through opposing actions of *O*-GlcNAc transferase (OGT) that transfers the GlcNAc group, and *O*-GlcNAcase (OGA) that removes it. Also, like its unsweetened counterpart, *O*-GlcNAcylation controls fundamental processes, e.g., protein fate, chromatin topology, DNA demethylation, and the circadian clock. Deregulation of the mechanisms controlling *O*-GlcNAc dynamics may be involved in the development of cancers, neuronal disorders such as Alzheimer’s disease, and metabolic conditions such as diabetes (1, 2).

This E-Book, which gathers Original Research papers, Method Articles, and Reviews published as part of a Research Topic in Frontiers in Endocrinology, is the opportunity to celebrate the thirtieth anniversary of the discovery of “*O*-GlcNAc.”

Honor to whom honor is due, it is to Gerald W. Hart, the discoverer of *O*-GlcNAc (3), that was assigned the task of writing a historical “Perspective” (4) as an introduction to this “Research Topic.”

Protein *O*-GlcNAcylation levels in cells, resulting from the opposing actions of OGT and OGA, are tightly regulated. Most people working in the field have experienced the now commonplace observation that manipulating cellular *O*-GlcNAc levels using drugs, siRNA or cDNA transfection results in counter-regulatory modification in OGT and OGA expression. However, no study had been specifically dedicated to investigate this question. In an original paper by Zhang et al. (5) the effect of a potent and highly selective OGA inhibitor, Thiamet-G, on OGT and OGA mRNA and protein levels, was systematically studied in different cell types. The authors observed that OGA is more sensitive than OGT to *O*-GlcNAc levels. Increases in OGA expression were not due to stabilization of OGA mRNA or protein, suggesting regulation of OGA mRNA via transcription, through as yet unknown mechanisms.

*O*-GlcNAcylation is generally presented as a glycosylation that occurs only in the cytosol, the nucleus, and to a lesser extent, in mitochondria, in contrast to “classical” and complex *N*- and *O*-glycosylations that take place in the endoplasmic *reticulum* and the Golgi apparatus, and that modify transmembrane, secreted and organelle-confined proteins. However, biology is often made of exceptions to rules, and *O*-GlcNAcylation of protein extracellular domains has been demonstrated in *Drosophila* (6). In this Research Topic, Nagnan-Le Meillour et al. (7) provide original data indicating that olfactory binding proteins (OBPs) secreted in pig nasal mucus are also modified by *O*-GlcNAc. They identified and cloned a conserved eOGT (EGF domain-specific OGT) in *Sus scrofa* and proposed that *O*-GlcNAcylation of OBPs could finely modulate their binding specificities for odors and pheromones.

Increased *O*-GlcNAcylation is involved in insulin resistance associated with diabetes and obesity (2). The adipose cell plays a central role in the regulation of energy homeostasis, in particular, through its capacity to secrete adipokines that modulate insulin sensitivity and pro-inflammatory cytokines. Wollaston-Hayden et al. (8) show that *O*-GlcNAc modulates the transcript levels of multiple secreted proteins in rodent adipocytes, and propose that *O*-GlcNAcylation of transcription factors such as Sp1 plays a role in adipokines gene transcription during insulin resistance.

Whereas OGT or OGA knock down is lethal in higher eukaryotes, *ogt1* and *oga1* null *C. elegans* are viable. Taking advantage of this model organism, Ghosh et al. (9) investigated the consequences of OGT or OGA ablation, and showed that disruption in *O*-GlcNAc cycling alters nucleotide sugar production, overall glycan composition and transcription of genes encoding key members of the HBP pathway.

Although more than 1000 proteins are already known targets for *O*-GlcNAcylation, it is likely that numerous *O*-GlcNAcylated proteins remain to be identified. In addition, one of the fascinating features of *O*-GlcNAc is its complex interplay with phosphorylation (1), either through regulation of phosphorylation at adjacent sites or by direct competition between *O*-GlcNAcylation and phosphorylation for the same site (the so-called Yin–Yang mechanism). In a Methods article, Cieniewski-Bernard et al. (10) describe the development of a multiplex, fluorescence-based proteomic strategy that permits to detect *O*-GlcNAcylated proteins, phosphoproteins, and the whole proteome on the same bi-dimensional gel.

This Research Topic also includes a number of reviews on some of the important biological and pathophysiological questions linked to *O*-GlcNAcylation. The perturbation of the *O*-GlcNAc cycle recently appeared as a hallmark of cancer cells (11). Jóźwiak et al. (12) review the role of *O*-GlcNAc in metabolic reprograming of cancer cells, through modification of metabolic enzymes, signaling proteins, and transcription factors, and Chaiyawat et al. (13) discuss these alterations specifically in breast and colorectal cancers. Epigenetic alterations also characterize numerous tumors, and recent data reviewed by Dehennaut et al. (14) reveal the involvement of *O*-GlcNAcylation as an epigenetic mark, and its role in chromatin remodeling and DNA methylation.

Numerous studies have provided evidence that *O*-GlcNAc negatively regulates insulin signaling (2), highlighting a link between hyperglycemia, insulin resistance, and glucotoxicity. Zhang et al. (15) review the implication of *O*-GlcNAcylation of signaling components and transcription factors in normal liver metabolism and in liver diseases, including insulin resistance, non-alcoholic fatty liver disease, and non-alcoholic steatohepatitis.

In a mini review, Benahmed et al. (16) also discuss the role of transcription factors in the control of energy metabolism, and more specifically the antagonistic relationships between ChREBP, which controls the expression of glycolytic and lipogenic genes, and the nuclear receptor FXR, which controls bile acid metabolism involved in gut–liver homeostasis. Interestingly, both transcription factors are modified by *O*-GlcNAcylation, although the consequences of this modification on ChREBP–FXR interaction remain to be explored.

Finally, as several lines of evidence indicate that *O*-GlcNAcylation regulates immune processes and may participate in hyperglycemia-associated inflammation, Baudoin and Issad (17) review the pro- and anti-inflammatory effects of *O*-GlcNAc, which may appear contradictory depending on cell types and pathophysiological situations. The field reviewed by these authors illustrates the complexity of signaling pathway regulation by *O*-GlcNAcylation. The control of inflammatory processes by *O*-GlcNAcylation is one of the innumerable *Terra incognita* to be explored.

Although not all aspects of *O*-GlcNAc biology could be presented in the Research Topic, we hope that it will excite the curiosity and stimulate the interest of young scientists for this ever-expanding, fascinating field.

## Conflict of Interest Statement

The authors declare that the research was conducted in the absence of any commercial or financial relationships that could be construed as a potential conflict of interest.
